# Direct neural transmission of vCJD/BSE in macaque after finger incision

**DOI:** 10.1007/s00401-020-02231-w

**Published:** 2020-10-06

**Authors:** Jacqueline Mikol, Jérôme Delmotte, Dolorès Jouy, Elodie Vaysset, Charmaine Bastian, Jean-Philippe Deslys, Emmanuel Comoy

**Affiliations:** Université Paris-Saclay, CEA, Service d’Etude des Prions et des Infections Atypiques, 18 Route du Panorama, 92265 Fontenay-aux-Roses, France

Non-human primates appeared as the closest model to study human iatrogenic prion diseases [14]: we report here the consequences of variant Creutzfeldt–Jakob disease/bovine spongiform encephalopathy (vCJD/BSE) inoculation in a cynomolgus macaque finger, with the demonstration of an original mode of propagation and the practical risk for professional exposure.

The distal right middle finger handpad of a 4-year-old macaque was incised on both lateral sides to induce local inflammation, and then injected with the equivalent of 10 mg of a BSE, orally challenged macaque brain [18]. After an 18 months period of finger clumsiness, the clinical disease (behaviour abnormalities, fear, hyperesthesia, gait disturbances, shaking) began 7.5 years after inoculation and euthanasia took place 2 months later for welfare reasons. Motor conduction velocity of the right median nerve was reduced to one-third of the left counterpart and sensory potential was not detected.

Histological and biochemical studies were performed as previously described. All the elements of the triad were present [7–9]: spongiform change was moderate in neocortex, striatum, brain stem, mild in spinal cord but severe in thalamus and cerebellum; neuronal loss was globally moderate, but severe in cerebellum and sacral spinal cord (vacuolated neurons); gliosis was severe in thalamus, cerebellum and brain stem and moderate elsewhere (Supplementary Fig. 1). ELISA and western blot (WB) showed the expected accumulation of PrP^res^ with BSE glycophoretic pattern at all levels of brain and spinal cord (Supplementary Fig. 2).

In the brain, PrP^d^ deposits were laminar into the cortical deep layers, massive into thalamus, basal ganglia, cerebellum, and brain stem. In spinal cord, PrP^d^ was symmetrically distributed, intense in the *Substantia gelatinosa* and nucleus dorsal of Clarke while decreased at sacral level. Deposits were diverse into the whole CNS: synaptic, perineuronal, reticular aggregates, mini-plaques, plaques, and incomplete florid plaques. The retinal plexiform layers were labelled (Supplementary Fig. 1i). There were no amyloid or tau deposits.

Unusual PrP^d^ deposits were observed along dendrites, short and long axons, neuritic threads tracing fine networks of straight lines or like strings of pearls (Supplementary Fig. 3). They were present into deep neocortex, basal ganglia, and motoneurons. Such long processes are not frequent but have been reported in human [13] and experimental studies [10, 22]. PrP^d^ deposits were also noted as very mild into striato-pallidal projections, both limbs of internal capsule and fornix (Supplementary Fig. 3). The presence of PrP^d^ in white matter has been reported (Supplementary text 4).

Peripherally, the expected PrP^d^ was undetectable in lymphoid organs, including spleen, through biochemical or immunohistochemical analyses, while prion replication was detected in the peripheral nervous system (PNS): PrP^d^ staining was visualized in many dorsal root ganglia (DRG) but only in nerves innervating the forelimb site of injection (median and ulnar nerves). At the cellular level, PrP^d^ was limited to ganglia and satellite cells in DRG and Schwann cells (Scs) all along nerves whereas axons were never labelled (Fig. 1). Previously, using postmortem immunohistochemical studies (listed in Supplementary text 5), PrP^d^ has been shown in peripheral nervous system in all forms of human neuropathies, albeit more frequently in vCJD, mostly in posterior root nerve fibres at adaxonal location and/or in ganglion and satellite cells. The restricted amount of PrP^d^ was repeatedly underlined but, recently, prion RT-QuiC was positive in all nerves examined [2]. PrP^d^ has also been described, first in scrapie [17] then in BSE, as limited “adaxonal deposits” or/and Sc deposits, with or without DRG cell involvement (review in [4] and Supplementary text 6). Previous studies of the mode of propagation of PrP^d^ have reported variable observations and analyses depending on strains, host species and genotype (Supplementary text 6); the authors discussed the role of the sensory route of trafficking of prions, the modifications of axonal transport, the centrifugal versus centripetal spread of PrP^d^.

**Fig. 1:**
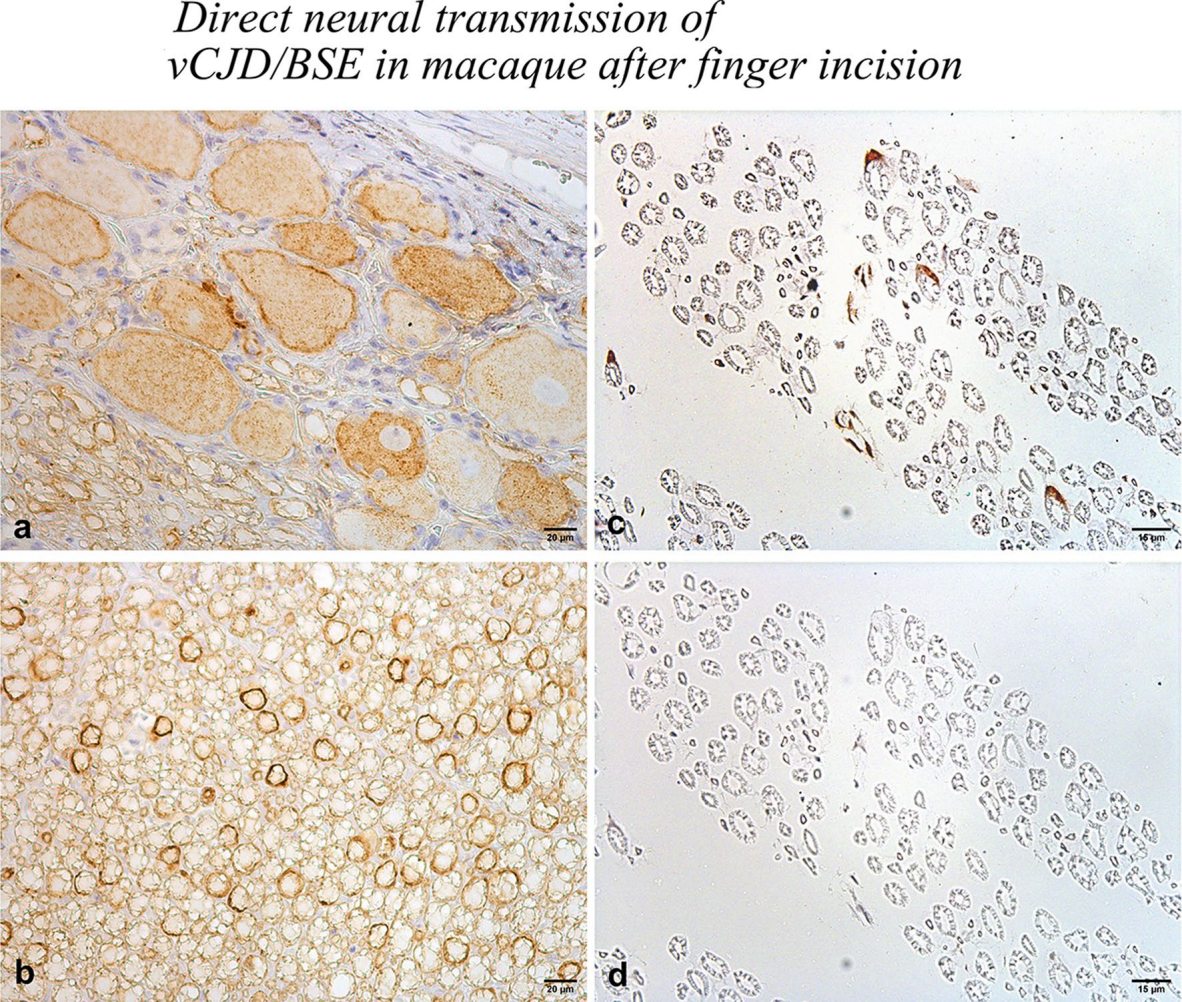
PrP^d^ immunostaining (SHA31) of lumbar root ganglia (**a**) (cytoplasmic dots in ganglia cells and larger aggregates in satellite cells) and lumbar proximal root (**b**) (adaxonal, but not axonal labelling). PrP^d^ immunostaining (3F4) of right proximal median nerve showing a few labelled Schwann cells (**c**). Isotype control (**d**)

After peripheral infection, accumulation of infectious agent is reputed to occur in lymphoid tissues before direct neuroinvasion [18, 19], even with very little apparent peripheral lymphoreticular deposition [6, 20]. Here, there is no apparent replication/amplification of vCJD/BSE agent in the lymphoid tissues of the exposed macaque. In this model, the neural contamination occurred directly in the highly innervated finger while neuroinvasion appears to occur in Scs along the median nerve to the DRG, with the appearance of the classical labelling of ganglion cells which indicates the onset of the first level of neuronal infection. This model provides direct evidence of the hypothesis of a sequential infection of Scs from the periphery to the CNS, followed by a secondary diffusion into the spinal cord, as already considered by our group [15] and others [1, 3, 11, 12, 21]. It is to note that studies based on intra-sciatic nerve injections in hamsters [16] and transgenic mice [12] had established a rate of transport of infectivity of, respectively, 0.5–2 mm and 0.7 mm per day. This key role of Scs could explain both the low speed of propagation and the discrepancy between the paucity of PrP^d^ into the distal part of the sensory nerves followed by the positivity of DRG, satellite cells and proximal roots.

In conclusion, we have observed that the exposure of a primate to vCJD/BSE through a distal finger lesion induces, after more than 7.5 years of silent incubation, a massive deposit of PrP^d^, strictly restricted to the nervous system and the eye.

Our data suggest a new type of pure unique peripheral nervous contamination in which the Scs would have a major role in the mode of centripetal progression of PrP^d^ in the peripheral nervous system. Moreover, considering the fact that, recently, “a variant CJD diagnosed 7.5 years after occupational exposure” (cryomicrotomy) in a technician was observed [5], this experimental case report supports the risk linked to professional exposure and reinforces the necessity of adequate measures of prevention.

## Electronic supplementary material

Below is the link to the electronic supplementary material.

Supplementary file1 (DOCX 18789 kb)
