# Supplementary material for: Direct neural transmission of vCJD/BSE in macaque after finger incision
Source: Acta Neuropathol. 2020 Oct 6;141(1):119–22. doi: 10.1007/s00401-020-02231-w (PMC7785535; doi:10.1007/s00401-020-02231-w)

**Supplementary text 4**

The presence of PrP^d^ in the white matter has been considered as a distinctive feature of multiple inherited prion diseases [27] or due to modifications of oligodendroglial cells [4], hypothesis ruled out by Prinz [25] but reinforced by the demonstration of PrP^d^ accumulation in oligodendroglial inner mesaxons [14].

**Supplementary text 5**

In few previous clinical cases, neuropathies have been observed, but detailed neuropathological studies were not totally convincing (review in [17]). As soon as 1989, Guiroy et al [9] reported labeling of numerous neurons, dystrophic axons and satellite cells in the trigeminal ganglia (TG) of 2 CJD cases. The extension of the disease process along the axons, either centripetally or centrifugally was questioned. Using anti-PrP antibodies, Hainfellner and Budka detected discrete PrP^d^ deposits, in a few posterior root nerve fibers “in an adaxonal location in one of nine CJD and the one GSS patients examined” [11]. The peripheral nerves were segmentally demyelinated but free of PrP^d^ deposition in the patient studied by Kovacs [17]. The involvement of the sympathetic nervous system was only shown in vCJD [10]. Subsequently, Head et al also demonstrated by immuno-histochemical analysis the involvement of TG (5/7 sCJD - 8/8 vCJD) and DRG (3/6 sCJD - 6/6 vCJD) but the absence of accumulation of PrP^d^ in peripheral nerves [12]; complementary WB analysis gave less positive results. Lee et al (2005), studying 20 specimens from diverse subgroups of CJD, detected PrP^d^ in 15 of 22 TG [21]. The staining was more intense in the ganglion cells in 2 vCJD with dark-membrane associated deposits and diffuse cytoplasmic labeling and a small number of satellite cells. Deposits were smaller in 2 iatrogenic CJD and 13 sporadic CJD (sCJD). In 9 DRG, deposits were seen at the periphery of the cells leading to infer the role of the sensory route in the trafficking of PrP^d^. In one case of sCJD [5], PrP^d^ was evidenced in the DRG and the superficial peroneal nerve. With histological observations very close to ours, Mead et al showed PrP^d^ around DRG ganglion cells, peripheral and cranial roots and multiple nerve fibres (illustrated in the median nerve Fig:5- H and I) in a familial prion disease, linked to PNRPY163X truncation mutation [23]. Screening 3 large cohorts, Kresl et al observed coarse aggregates within the extra-cerebral portion of the vagus nerve in the VV2 CJD cases and fine dots along the nerve, within the axons surrounded by myelin sheaths, in genetic forms [18].

**Supplementary text 6**

Experimentally, many studies have converged to suggest that PNS may be the final common pathway for neuroinvasion in vivo. The first results were obtained using sheep scrapie strains. Besnoit had first described the “tremblante” as a “névrite périphérique enzootique du mouton” [3]. The pivotal studies were performed by Kimberlin et al [15, 16] who showed that inoculation under the peri-neurium of the sciatic nerve led to the direct spread of infection to brain, by-passing the need for extra-neural replication. Using biochemical approaches (Western blot), Glatzel and Aguzzi showed that transgenic mice overexpressing PrP^c^ (tga20), undergo rapid neuroinvasion upon intra-sciatic nerve and footpad inoculation of prions [7]. No accumulation of PrP^d^ was visualized in their spleens, as observed in our monkey and the studies of Lasmézas et al [19] and Race et al [26]. At the opposite, wild type mice accumulated splenic PrP^d^ and prion infectivity titers of sciatic nerve were much lower than in tg mice. Subsequently, the same group described prion neuroinvasion within the sympathetic nerve and questioned the role of Scs [20]. In 1999, using immunocytochemistry, Groschup et al visualized granular PrP^d^ deposits within TG, DRG and sympathetic ganglions, more precisely in ganglion and satellite cells and along the adjacent axons in sheep and hamsters [8]. All the conceivable modes of spread of prions in the PNS were reviewed by Beekes and McBride [2].

The second period was observed after recognition of classical and atypical H (High- type) and L (Low- type) BSE affected cattle. In cBSE [22] and L-BSE [13], PrP^d^ was detected, using WB, in DRG and peripheral nerve trunks. In these L-BSE cows, among the large number of ganglia, cranial and peripheral nerves investigated, increasing positivity was demonstrated in a time-dependent manner and positive control obtained after transmission to Tg mice. Using bovine PrP transgenic mice and a PMCA protocol, PrP^d^ was found in cattle TG and DRG [6]. Okada et al, reproducing the same experimental study as his co-author Iwamaru, showed in H-type BSE deposits in TG, multiple DRG, satellite cells and nerves [24]. It was to note the presence of PrP^d^ granules mainly in the Scs and fibroblast-like cells. Recently, in a large series, including all forms of BSE, Balkema-Buschmann et al also showed the involvement of the PNS (TG, DRG), and reported that H -BSE seems to spread rapidly from the CNS into the periphery via the glial system such as Scs [1]. The hypothesis of a centrifugal spread was related to the observation of infectivity in the late stages of both atypical forms.

**Supplementary references**

1. Balkema-Buschmann A, Priemer G, Ulrich R, Strobelt R, Hills B, Groschup MH (2019) Deciphering the BSE-type specific cell and tissue tropisms of atypical (H and L) and classical BSE. Prion 13:160–172. doi: 10.1080/19336896.2019.1651180

2. Beekes M, McBride PA (2007) The spread of prions through the body in naturally acquired transmissible spongiform encephalopathies. FEBS J 274:588–605. doi: 10.1111/j.1742-4658.2007.05631.x

3. Besnoit, Ch (1899) La tremblante ou névrite périphérique enzootique du mouton. Rev Vet 265–277

4. El Hachimi KH, Chaunu M-P, Brown P, Foncin J-F (1998) Modifications of Oligodendroglial Cells in Spongiform Encephalopathies. Exp Neurol 154:23–30. doi: 10.1006/exnr.1998.6894

5. Favereaux A, Quadrio I, Vital C, Perret-Liaudet A, Anne O, Laplanche J-L et al. (2004) Pathologic Prion Protein Spreading in the Peripheral Nervous System of a Patient With Sporadic Creutzfeldt-Jakob Disease. Arch Neurol 61:747–750. doi: 10.1001/archneur.61.5.747

6. Franz M, Eiden M, Balkema-Buschmann A, Greenlee J, Schatzl H, Fast C et al. (2012) Detection of PrP(Sc) in Peripheral Tissues of Clinically Affected Cattle After Oral Challenge With Bovine Spongiform Encephalopathy. J. Gen. Virol. 2740–2748. doi: 10.1099/vir.0.044578-0.

7. Glatzel M, Aguzzi A (2000) PrPC expression in the peripheral nervous system is a determinant of prion neuroinvasion. J Gen Virol 81:2813–2821. doi: 10.1099/0022-1317-81-11-2813

8. Groschup MH, Beekes M, McBride PA, Hardt M, Hainfellner JA, Budka H (1999) Deposition of disease-associated prion protein involves the peripheral nervous system in experimental scrapie. Acta Neuropathol (Berl) 98:453–457. doi: 10.1007/s004010051108

9. Guiroy DC, Shankar SK, Gibbs CJ, Messenheimer JA, Das S, Gajdusek DC (1989) Neuronal degeneration and neurofilament accumulation in the trigeminal ganglia in creutzfeldt-jakob disease. Ann Neurol 25:102–106. doi: 10.1002/ana.410250119

10. Haïk S, Faucheux BA, Sazdovitch V, Privat N, Kemeny J-L, Perret-Liaudet A et al. (2003) The sympathetic nervous system is involved in variant Creutzfeldt-Jakob disease. Nat Med 9:1121–1122. doi: 10.1038/nm922

11. Hainfellner JA, Budka H (1999) Disease associated prion protein may deposit in the peripheral nervous system in human transmissible spongiform encephalopathies. Acta Neuropathol (Berl) 98:458–460. doi: 10.1007/s004010051109

12. Head MW, Ritchie D, Smith N, McLoughlin V, Nailon W, Samad S et al. (2004) Peripheral tissue involvement in sporadic, iatrogenic, and variant Creutzfeldt-Jakob disease: an immunohistochemical, quantitative, and biochemical study. Am J Pathol 164:143–153. doi: 10.1016/S0002-9440(10)63105-7

13. Iwamaru Y, Imamura M, Matsuura Y, Masujin K, Shimizu Y, Shu Y et al. (2010) Accumulation of L-type Bovine Prions in Peripheral Nerve Tissues. Emerg Infect Dis 16:1151–1154. doi: 10.3201/eid1607.091882

14. Jeffrey M, Goodsir C, McGovern G, Barmada SJ, Medrano AZ, Harris DA (2009) Prion Protein with an Insertional Mutation Accumulates on Axonal and Dendritic Plasmalemma and Is Associated with Distinctive Ultrastructural Changes. Am J Pathol 175:1208–1217. doi: 10.2353/ajpath.2009.090125

15. Kimberlin RH, Hall SM, Walker CA (1983) Pathogenesis of mouse scrapie: Evidence for direct neural spread of infection to the CNS after injection of sciatic nerve. J Neurol Sci 61:315–325. doi: 10.1016/0022-510X(83)90165-X

16. Kimberlin RH, Walker CA (1980) Pathogenesis of mouse scrapie: evidence for neural spread of infection to the CNS. J Gen Virol 51:183–187. doi: 10.1099/0022-1317-51-1-183

17. Kovács T, Arányi Z, Szirmai I, Lantos PL (2002) Creutzfeldt-Jakob Disease With Amyotrophy and Demyelinating Polyneuropathy. Arch Neurol 59:1811–1814. doi: 10.1001/archneur.59.11.1811

18. Kresl P, Rahimi J, Gelpi E, Aldecoa I, Ricken G, Danics K et al. (2019) Accumulation of prion protein in the vagus nerve in creutzfeldt–jakob disease. Ann Neurol 85:782–787. doi: 10.1002/ana.25451

19. Lasmezas CI, Cesbron JY, Deslys JP, Demaimay R, Adjou KT, Rioux R et al. (1996) Immune system-dependent and -independent replication of the scrapie agent. J Virol 70:1292–5.

20. Lasmezas CI, Fournier JG, Nouvel V, Boe H, Marce D, Lamoury F et al. (2001) Adaptation of the bovine spongiform encephalopathy agent to primates and comparison with Creutzfeldt-- Jakob disease: implications for human health. Proc Natl Acad Sci U S A 98:4142–7. doi: 10.1073/pnas.041490898

21. Lee C-CM, Kuo LT, Wang CH, Scaravilli F, An SF (2005) Accumulation of Prion Protein in the Peripheral Nervous System in Human Prion Diseases. J Neuropathol Exp Neurol 64:716–721. doi: 10.1097/01.jnen.0000175330.66715.08

22. Masujin K, Matthews D, Wells Ga, Mohri S, Yokoyama T (2007) Prions in the Peripheral Nerves of Bovine Spongiform Encephalopathy-Affected Cattle. J. Gen. Virol. 1850–1858. doi: 10;1099/vir.0.82779-0

23. Mead S, Gandhi S, Beck J, Caine D, Gajulapalli D, Carswell C et al. (2013) A novel prion disease associated with diarrhea and autonomic neuropathy. N Engl J Med 369:1904–14. doi: 10.1056/NEJMoa1214747

24. Okada H, Iwamaru Y, Yokoyama T, Mohri S (2013) Immunohistochemical Detection of Disease-Associated Prion Protein in the Peripheral Nervous System in Experimental H-Type Bovine Spongiform Encephalopathy. Vet Pathol 50:659–663. doi: 10.1177/0300985812471541

25. Prinz M, Montrasio F, Furukawa H, van der Haar ME, Schwarz P, Rülicke T et al. (2004) Intrinsic Resistance of Oligodendrocytes to Prion Infection. J Neurosci 24:5974–5981. doi: 10.1523/JNEUROSCI.0122-04.2004

26. Race R, Oldstone M, Chesebro B (2000) Entry versus Blockade of Brain Infection following Oral or Intraperitoneal Scrapie Administration: Role of Prion Protein Expression in Peripheral Nerves and Spleen. J Virol 74:828–833. doi: 10.1128/jvi.74.2.828-833.2000

27. Reiniger L, Mirabile I, Lukic A, Wadsworth JD, Linehan JM, Groves M et al. (2013) Filamentous white matter prion protein deposition is a distinctive feature of multiple inherited prion diseases. Acta Neuropathol Commun 1. doi: 10.1186/2051-5960-1-8

**Supplementary figures legends**

**Supplementary fig. 1**

**(a)** Spongiform change in nucleus caudatus (Hematoxylin-Eosin: H&E). **(b)** Spongiform change in pulvinar (H&E). **(c)** sacral spinal cord vacuolated neurons (H&E). **(d)** Immunostaining with monoclonal anti-PrP antibody 3F4, showing, lenticular neuronal labeling and, at higher magnification, aggregates along the dendrite trunks **(e)** or thalamic synaptic deposits labelled with Sha-31 **(f).** Spongiform change of the cerebellum (H&E) **(g)** and labeling with monoclonal anti-PrP antibody 1802Y **(h).** PrP^d^ immunostaining with monoclonal anti-PrP antibody SHA31 of the two plexiform layers of the retina **(i)**.

**Supplementary fig. 2**

PrPres from cerebral (frontal and occipital), cerebellar and spinal cord (7^th^ cervical and 10^th^ dorsal metamers) samples was purified with Bio-Rad TeSeE kit, and then detected with either Bio-Rad TeSeE detection kit or by western blotting using monoclonal anti-PrP antibody Sha-31. Serial dilutions of the purified inoculum injected in the finger of the macaque (i.e. parietal cortex from a BSE-infected macaque) were used as references. Frontal cortex was tested 1:3 diluted, occipital cortex and cerebellum were tested 1:10 diluted (spinal cord samples were tested neat). Both techniques have similar limits of detection (1 μg of terminally BSE-affected macaque brain, i.e. 10^-6^/g).

**Supplementary fig. 3**

Neuritic processes appearing as “strings of pearls” (**a** and **b**: higher magnification of a). **(c)** Globus pallidus nucleus labeled with monoclonal anti-PrP antibody 3F4. **(d)** Parietal cortex labeled with monoclonal anti-PrP antibody 3F4 or **(e)** 1802Y. **(f)** white matter striato-pallidal tracts labeled with 3F4.


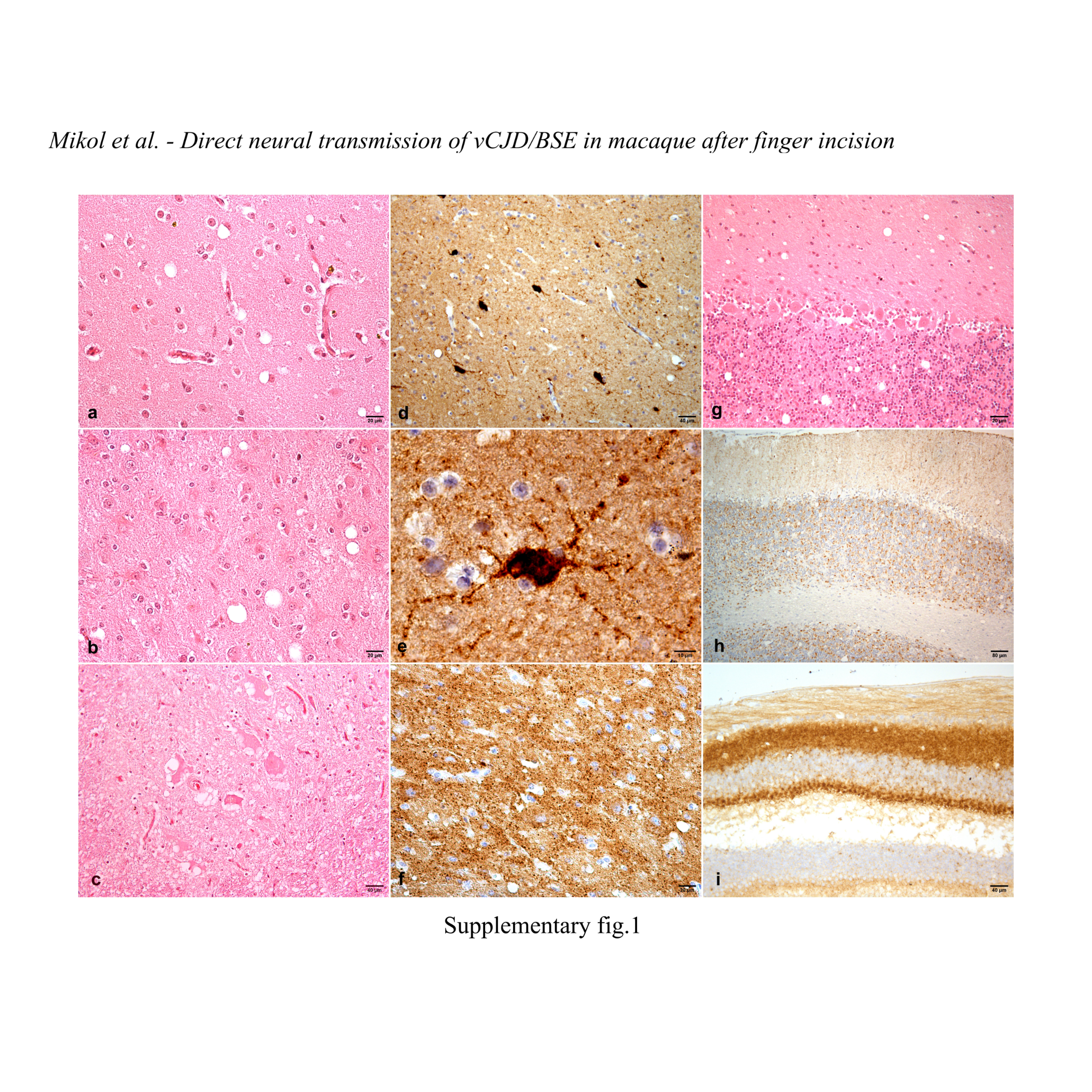


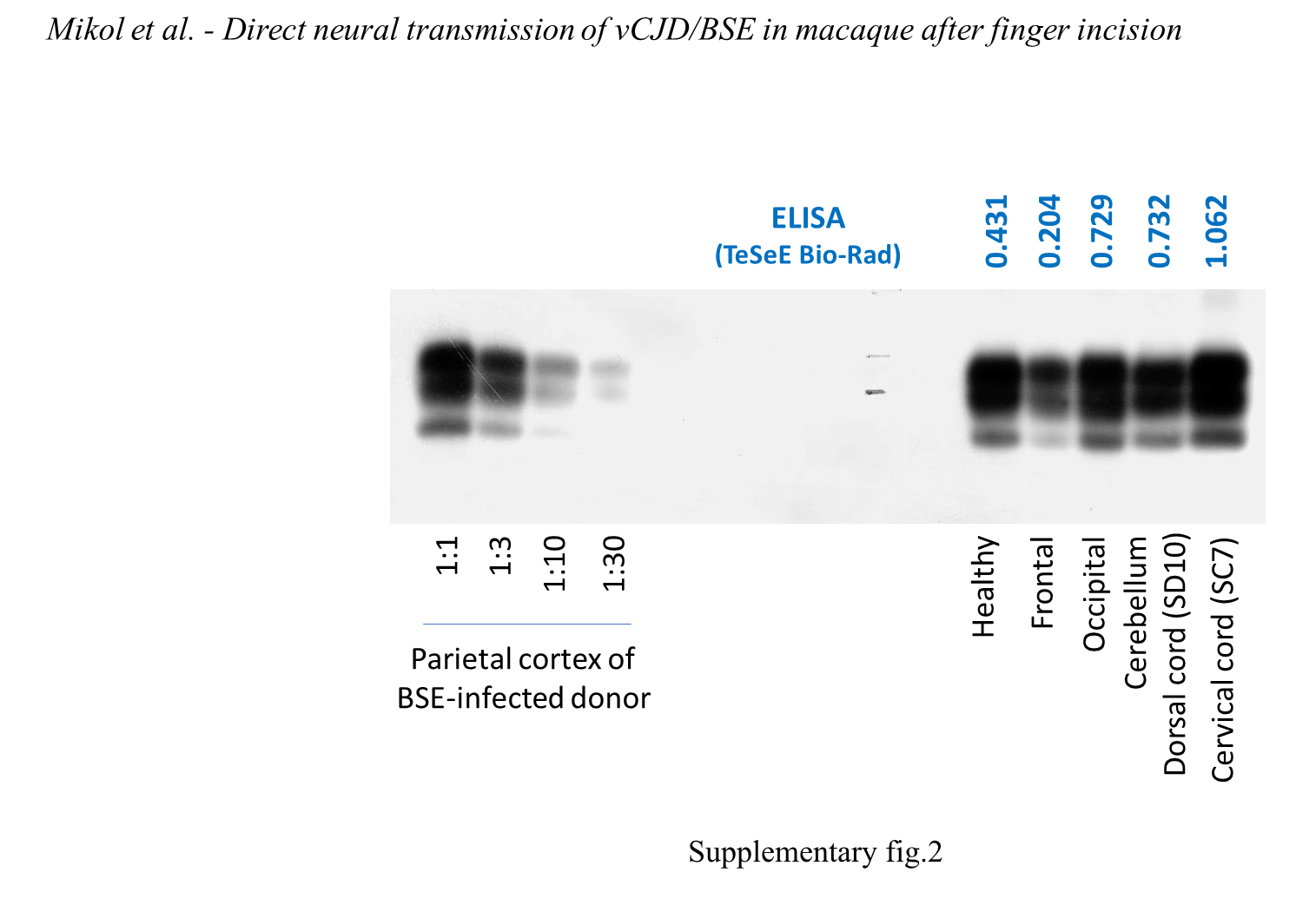


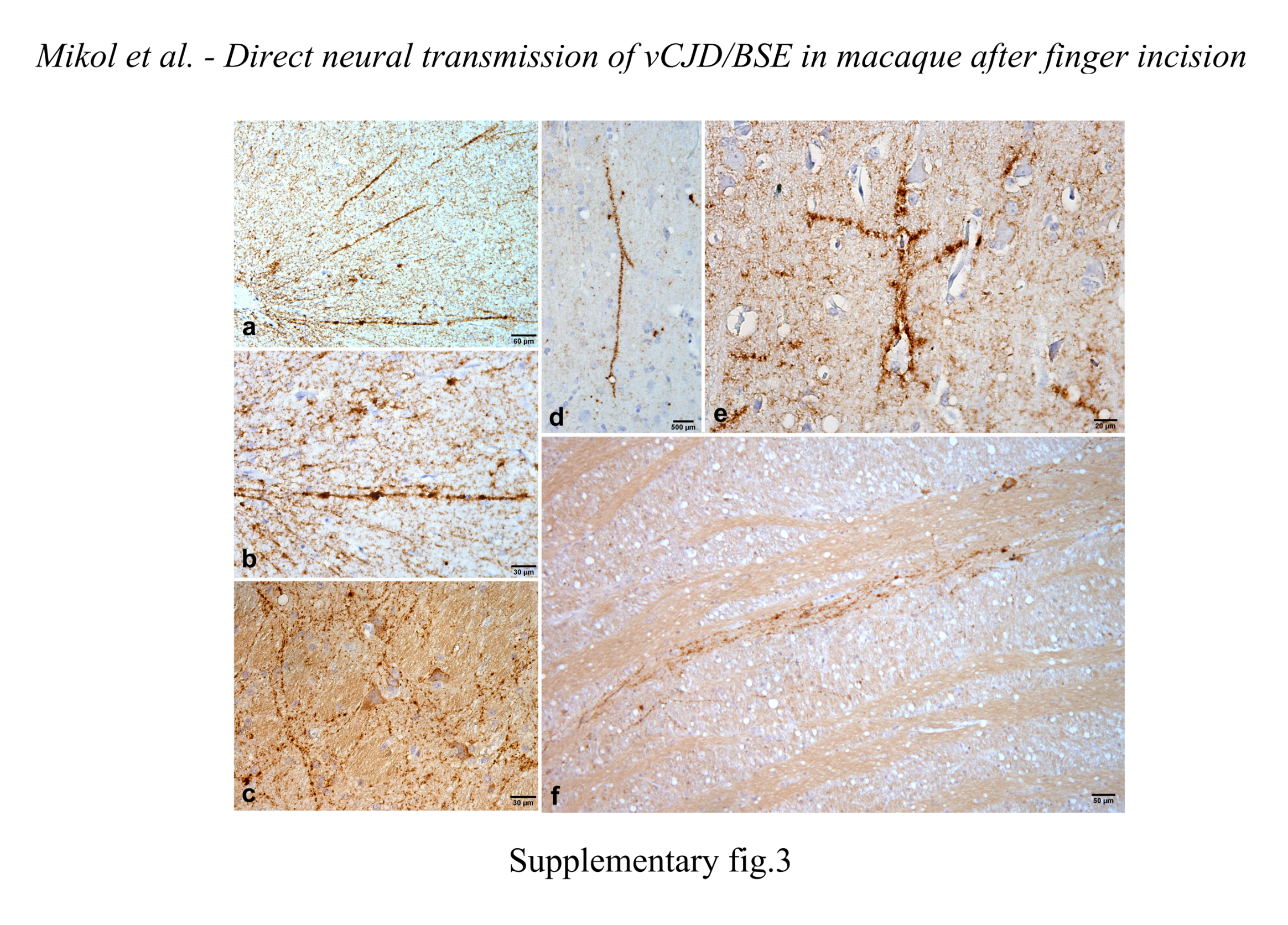

Supplement: Supplementary file 1 — Supplementary file1 (DOCX 18789 kb) [file 401_2020_2231_MOESM1_ESM.docx]
